# Effects of glutamine deprivation on oxidative stress and cell survival in breast cell lines

**DOI:** 10.1186/s40659-019-0224-9

**Published:** 2019-03-27

**Authors:** Mokgadi Violet Gwangwa, Anna Margaretha Joubert, Michelle Helen Visagie

**Affiliations:** Department of Physiology, Faculty of Health Sciences, University of Pretoria, Private Bag X323, Arcadia, Pretoria, 0007 South Africa

**Keywords:** Glutamine deprivation, ROS, Mitochondrial membrane potential, Cell cycle progression, Apoptosis

## Abstract

**Background:**

Tumourigenic cells modify metabolic pathways in order to facilitate increased proliferation and cell survival resulting in glucose- and glutamine addiction. Previous research indicated that glutamine deprivation resulted in potential differential activity targeting tumourigenic cells more prominently. This is ascribed to tumourigenic cells utilising increased glutamine quantities for enhanced glycolysis- and glutaminolysis. In this study, the effects exerted by glutamine deprivation on reactive oxygen species (ROS) production, mitochondrial membrane potential, cell proliferation and cell death in breast tumourigenic cell lines (MCF-7, MDA-MB-231, BT-20) and a non-tumourigenic breast cell line (MCF-10A) were investigated.

**Results:**

Spectrophotometry demonstrated that glutamine deprivation resulted in decreased cell growth in a time-dependent manner. MCF-7 cell growth was decreased to 61% after 96 h of glutamine deprivation; MDA-MB-231 cell growth was decreased to 78% cell growth after 96 h of glutamine deprivation, MCF-10A cell growth was decreased 89% after 96 h of glutamine deprivation and BT-20 cell growth decreased to 86% after 24 h of glutamine deprivation and remained unchanged until 96 h of glutamine deprivation. Glutamine deprivation resulted in oxidative stress where superoxide levels were significantly elevated after 96 h in the MCF-7- and MDA-MB-231 cell lines. Time-dependent production of hydrogen peroxide was accompanied by aberrant mitochondrial membrane potential. The effects of ROS and mitochondrial membrane potential were more prominently observed in the MCF-7 cell line when compared to the MDA-MB-231-, MCF-10A- and BT-20 cell lines. Cell cycle progression revealed that glutamine deprivation resulted in a significant increase in the S-phase after 72 h of glutamine deprivation in the MCF-7 cell line. Apoptosis induction resulted in a decrease in viable cells in all cell lines following glutamine deprivation. In the MCF-7 cells, 87.61% of viable cells were present after 24 h of glutamine deprivation.

**Conclusion:**

This study demonstrates that glutamine deprivation resulted in decreased cell proliferation, time-dependent- and cell line-dependent ROS generation, aberrant mitochondrial membrane potential and disrupted cell cycle progression. In addition, the estrogen receptor positive MCF-7 cell line was more prominently affected. This study contributes to knowledge regarding the sensitivity of breast cancer cells and non-tumorigenic cells to glutamine deprivation.

**Electronic supplementary material:**

The online version of this article (10.1186/s40659-019-0224-9) contains supplementary material, which is available to authorized users.

## Background

Tumorigenic cells possess altered metabolism that supports the high energy- and nutrient production required for hyperproliferation and increased cell survival compared to non-tumorigenic- and differentiated cells. Since glutaminolysis is an alternative pathway for energy production, elevation in glutamine consumption and subsequent glutamine addiction is frequently observed in cancer metabolism [1, 2].

Glutamine is crucial for bioenergetic processes and biosynthetic needs [3]. Glutamine provides carbon and nitrogen required for the production of energetic-, biosynthetic- and reductive precursors in tumorigenic cells including deamination of glutamine and subsequent decarboxylated to produce α-ketoglutarate which enters the Krebs cycle and produces adenosine triphosphate (ATP) [4, 5]. Thus, due to the aberrant tumorigenic metabolic requirements and an increased need for carbon sources, tumorigenic cells become glutamine-addicted [6]. Furthermore, glutamine is converted to glutamate by mitochondrial glutaminase which has been reported to be upregulated in tumourigenic cells and is required for proliferation of several tumourigenic cell lines including human lymphoid tumour cell line and prostate cancer cell line (PC3) [7, 8]. B lymphocyte mouse hybridoma cells (Sp2/0) cultured in glutamine concentrations ranging from 0.1 to 1 mM in 24 h demonstrated a dose-dependent decrease in cell viability and increased induction of apoptosis. Furthermore, glutamine deprivation induced apoptosis in RIE-1 cells, SP2/0-At14 cells and HL60 cells [9]. Glutamine deprivation also enhanced cell death in tumour necrosis factor related apoptosis inducing ligand (TRAIL)-induced apoptosis in human breast cancer cells (MDA-MB-231) by inducing death receptor 5 expression and endoplasmic reticulum stress [10]. The protective effects of glutamine against apoptosis induction was verified when glutamine supplementation reduced the apoptosis induction triggered by heat shock, irradiation and c-Myc overexpression [11–13]. Furthermore, the antiapoptotic activity of glutamine is reportedly independent from its utilization as an energy source indicating that glutamine plays an essential role in signaling pathways relating to cell survival [9, 14].

Aerobic glycolysis and mitochondrial oxidative phosphorylation are cellular sources of reactive oxygen species (ROS) which are fundamental signaling molecules at low concentrations. ROS includes hydroxyl radical (OH·), hydrogen peroxide (H_2_O_2_) and superoxide (O_2_^−^) [15]. Approximately 1–5% of oxygen is converted into superoxide when electrons leak from the electron transport chain and are transferred to oxygen [16]. However, under stress conditions, additional electrons prematurely leak from the respiratory chain which will exacerbate superoxide production, thus causing detrimental effects which may include apoptosis [15]. High ROS quantities can cause damage to macromolecules including DNA, triggering senescence and permeabilization of the mitochondria, leading to the release of cytochrome *c* which also induces apoptosis [17].

Glutamine is an abundant- and versatile nutrient that is involved in energy formation, redox homeostasis and signal transduction in cancer cells. In this study the influence of glutamine deprivation was investigated on cell proliferation, morphology, oxidative stress mitochondrial membrane potential and apoptosis induction in breast tumorigenic cell lines and a non-tumorigenic cell line. The knowledge pertaining to how sensitive tumorigenic- and non-tumorigenic cells are to glutamine deprivation will be crucial to future therapeutics targeting cancer cell metabolism.

## Materials and methods

### Cell lines

The MCF-7 cell line is an adherent tumorigenic luminal subtype A human breast cell line that is estrogen receptor (ER) positive, progesterone receptor (PR) positive and human epidermal growth factor receptor 2 (HER2) negative and was derived from a metastatic site [18, 19]. The MDA-MB-231 cells are adherent tumorigenic basal breast cells that are ER negative, PR negative and HER2 negative and were derived from a metastatic site [20]. BT-20 is an adherent tumorigenic basal subtype non-metastatic human breast cell that is ER negative, PR negative and HER2 negative [21]. The MCF-10A cell line is an adherent non-tumorigenic basal subtype human breast cell line that is ER negative, PR negative and HER2 negative. All cell lines were supplied by ATCC (Manassas, Virginia, United States of America) [22].

All four cell lines were cultured in were cultured in 75 cm^2^ tissue culture flasks at 37 °C and 5% CO_2_ atmospheric conditions. MCF-7- and MDA-MB-231 cells were propagated in Dulbecco’s minimum essential medium eagle (DMEM) supplemented with dialysed fetal calf serum (56 °C, 30 min), 100 U/ml penicillin G, 100 µg/ml streptomycin and fungizone (250 µg/l) [Sigma Chemical Co (St. Louis, Missouri, United States of America)] [19, 20]. BT-20 cells were cultured in growth medium consisting of a 1:1 mixture of DMEM and Ham’s-F12 medium, 10% heat-inactivated dialysed fetal calf serum (56 °C, 30 min), 100 U/ml penicillin G, 100 µg/ml streptomycin and fungizone (250 µg/l) [Sigma Chemical Co (St. Louis, Missouri, United States of America)] [21]. MCF-10A cells were cultured in growth medium consisting of a 1:1 mixture of DMEM and Ham’s-F12 medium, 20 ng/ml epidermal growth factor (EGF), 100 ng/ml cholera toxin, 10 µg/ml insulin and 500 ng/ml hydrocortisone, supplemented with 10% dialysed heat-inactivated fetal calf serum (56 °C, 30 min), 100 U/ml penicillin G, 100 µg/ml streptomycin and fungizone (250 µg/l) [Sigma Chemical Co (St. Louis, Missouri, United States of America)] [22]. This study used dialysed fetal calf serum uses a specialised process where the serum is exhaustively dialyzed against saline to ensure that glucose and glutamine had been removed while avoiding the precipitation of serum proteins.

### Reagents

All reagents were obtained from Sigma Chemical Co (St. Louis, Missouri, United States of America) except if otherwise specified. Crystal violet dye was provided by Merck (Pty) Ltd (Johannesburg, Gauteng, South Africa). Haematoxylin, eosin, propidium iodide (PI), dehydroethidium (DHE) and 2, 7-dichlorofluoresceindiacetate (DCFDA) were purchased from Sigma Chemical Co (St. Louis, Missouri, United States of America). The Annexin V-FITC antibody and Mitocapture™ was manufactured and obtained from Biolegend (San Diego, California, United States of America) and Biovision (Milpitas, California, United States of America) respectively.

The positive control for apoptosis induction and cell cycle progression included cells propagated in 2-methoxyestradiol-bis-sulfamate for 48 h. Negative controls for exposure conditions comprised of cells propagated in complete growth medium. Exposure condition/period referred to cells propagated in DMEM not containing glutamine, but supplemented with fetal calf serum, penicillin G, streptomycin and fungizone for the specified time period.

### Methods

#### Cell proliferation

##### Crystal violet (spectrophotometry)

Crystal violet staining was used to demonstrate the influence of glutamine deprivation on proliferation. Crystal violet allows for the quantification of cell number by intercalating with the DNA resulting in a purple colour. Crystal violet thus quantifies cell number in monolayer cultures as a function of the absorbance of the dye taken up by the cells [23].

Cells were plated in a 96 well plate at a density of 4000 cells per well. The plate was incubated overnight at 37 °C to allow for attachment. Cells were washed with phosphate-buffered saline (PBS) thrice and DMEM was replaced with DMEM not containing glutamine for 24 h, 48 h, 72 h and 96 h since literature has shown optimal antiproliferative activity at these time intervals [24]. Cells were fixed using 100 µl gluteraldehyde for 15 min at room temperature. Gluteraldehyde was removed and 100 µl crystal violet was added to each well. Plates were subsequently incubated for 30 min at room temperature and washed with water. The dye was solubilized with 200 µl triton X-100 and plates were left at room temperature for 30 min. The solubilized crystal violet solution (100 µl) was then aspirated into a new plate and absorbance was measured at 570 nm using an EPOCH Microplate Reader [Biotek Instruments, Inc. (Winooski, Vermont, United States of America)] [23].

#### Morphology

##### Haematoxylin and eosin (light microscopy)

Haematoxylin and eosin staining was used to evaluate the in vitro effects of glutamine starvation on morphology. Haematoxylin stains the nucleus blue and eosin stains the cytoplasm pink and thus also allows for the identification of interphase, the phases of mitosis and cells demonstrating elongation or characteristics of apoptosis [23].

Cells (200,000) were seeded onto heat-sterilized coverslips in a six well plate and left overnight. Cells were exposed to DMEM not containing glutamine as described previously in the crystal violet section. Coverslips were moved to staining dishes and treated with Bouin’s fixative for 30 min. The fixative was discarded and subsequently 70% ethanol was added to the coverslips and left for 20 min at 25 °C. Coverslips were washed with distilled water and haematoxylin was added. Samples were incubated at room temperature for 20 min and the haematoxylin solution was discarded. Coverslips were washed with distilled water followed by the addition of 70% ethanol for 5 min. Ethanol was discarded and 1% eosin was added and left for 5 min. The eosin was discarded and coverslip were rinsed with 70% ethanol, 96% ethanol and 100% ethanol and xylol twice for 5 min each. Coverslips were mounted on microscope slides using Entelan. Images were captured with a Zeiss Axiovert MRc microscope (Zeiss, Oberkochen, Germany) [23]. Quantitative data for mitotic indices were obtained by counting 1000 cells on each slide of the biological replicates (repeated three times) and data was expressed as a percentage of cells in each phase of interphase, mitosis (anaphase, metaphase and prophase) and cells demonstrating abnormal characteristics including cell elongation, abnormally large rounded cells, shrunken cells, membrane blebbing and apoptotic bodies.

#### Reactive oxygen species

##### Oxidative stress (flow cytometry)

The influence of glutamine deprivation on superoxide and hydrogen peroxide production was investigated as indicators of oxidative stress by means of DCFDA and DHE. Superoxide oxidizes HE to form a unique fluorescent compound, DHE, but not ethidium. DHE is oxidized by superoxide and not by hydroxyl radicals, singlet O_2_ or nitrogen radical. Hydrogen peroxide production was measured using DCFDA which is a non-fluorescence probe, is oxidized by hydrogen peroxide, hydroxyl radicals and peroxides. A fluorescent derivative, 2, 7-dichlorofluorescein, can be detected by with maximum excitation and emission spectra of 495 nm and 529 nm [20].

Cells (500,000) were seeded in T25 cm^2^ flasks and left overnight to allow for attachment. Cells were exposed to DMEM not containing glutamine as described previously in the crystal violet section. Cells were trypsinized and incubated in PBS (1 ml) containing 10 μM HE (15 min) or 20 μM DCFDA for (25 min). The fluorescent products (DCF and DHE) were measured with a FC500 System flow cytometer (Beckman Coulter South Africa (Pty) Ltd) (Pretoria, Gauteng, South Africa) using CXP software (Beckman Coulter South Africa (Pty) Ltd) (Pretoria, Gauteng, South Africa).

#### Mitochondrial membrane potential

##### Mitocapture™ (flow cytometry)

The effect of glutamine deprivation on the mitochondrial membrane potential was investigated using Mitocapture™. The cationic dye, 5,5′,6,6′-tetrachloro-1,1′,3,3′-tetraethylbenzimidazolylcarbocyanine iodide is provided in the Mitocapture™ Apoptosis Detection Kit and fluoresces a bright red colour when bound to an undamaged cell whereas in apoptotic cells it fluoresces green due to accumulating in the nucleus [25]. Mitochondrial membrane potential can experience hyperpolarization where the membrane potential is predominately negative whilst depolarisation functions in the opposite manner. Hyperpolarisation has an inhibitory effect as the inner membrane becomes more negative [26].

Cells were seeded at 500,000 cells per T25 cm^2^ and left overnight. Cells were exposed to DMEM not containing glutamine as described previously in the crystal violet section. Harvested cells were centrifuged, resuspended in diluted Mitocapture solution (1 μl Mitocapture: 1 ml pre-warmed incubation buffer) and incubated for 20 min (room temperature). Cells were centrifuged and cells were resuspended in 1 ml pre-warmed incubation buffer (37 °C). Cells were analyzed with a FC500 System flow cytometer (Beckman Coulter South Africa (Pty) Ltd) (Pretoria, Gauteng, South Africa) as described previously.

#### Cell cycle progression

##### Propidium iodide staining (flow cytometry)

The effects of glutamine deprivation on the cell cycle progression were investigated by means of flow cytometry. PI stains the DNA and thus enables the quantification of DNA correlating with stages of the cell cycle during cell division [20].

Cells (500,000) were plated in T25 cm^2^ flask and left overnight. Cells were exposed to DMEM not containing glutamine as described previously in the crystal violet section. Subsequently, cells were trypsinized and resuspended in 1 ml PBS. Cells were centrifuged and cells were resuspended in ice-cold PBS (200 µl) containing 0.1% fetal calf serum. Ice-cold 70% ethanol (4 ml) was added in a dropwise manner and cells were stored at 4 °C for 24 h. Cells were centrifuged and resuspended in 1 ml PBS containing PI (40 µg/ml), RNAse A (100 µg/ml) and triton X-100 (0.1%) and incubated at 37 °C for 45 min. PI fluorescence was measured using a FC500 System flow cytometer (Beckman Coulter South Africa (Pty) Ltd) (Pretoria, Gauteng, South Africa) as described before.

#### Apoptosis induction

##### Apoptosis induction using Annexin V-fluorescein isothiocyanate (flow cytometry)

The influence of glutamine deprivation on induction apoptosis and necrosis were evaluated and quantified using flow cytometry in combination with Annexin V-fluorescein isothiocyanate (FITC) and PI [23]. Cells maintain constant division of several phospholipids between inner- and outer portions of the plasma membrane. This is regulated by phospholipid translocases that are lipid specific and require ATP for translocation. Phosphatidylserine is located in the inner membrane facing the cytosol [27]. In apoptosis, the calcium-dependent phospholipids scramblase activity is activated which results in the scrambling the aminophospholipids over the inner- and outer membrane. Subsequent loss of the plasma membrane organisation causes a flip action aided by an enzyme named flippase of the phosphatidylserine layer of the cell membrane thus providing Annexin V-FITC a binding site [28]. PI is membrane impermeable and thus only enters the cell when the membrane is compromised due to apoptosis or necrosis [28].

Cells (500,000) were seeded in T25 cm^2^ flasks and left overnight. Cells were exposed to DMEM not containing glutamine as described previously in the crystal violet section. Cells were trypsinized and resuspended in 1× binding buffer (1 ml). After centrifuging, cells were resuspended in 1× binding buffer (100 μl), Annexin V-FITC (2.5 μl) and samples were incubated for 15 min in the dark (room temperature). Cells were washed by adding 1× binding buffer (1 ml). After centrifugation, cells were resuspended in of 1× binding buffer (500 μl). Immediately prior to analysis, 12.5 μl of PI (40 µg/ml) was added and gently mixed. PI fluorescence and Annexin V-FITC fluorescence were analyzed with a FC500 System flow cytometer (Beckman Coulter South Africa (Pty) Ltd) (Pretoria, Gauteng, South Africa) as discussed previously.

## Statistics

Qualitative data were obtained from light microscopy. Quantitative data for mitotic indices were obtained by counting 1000 cells on each slide of the biological replicates (repeated three times) and data were expressed as a percentage of cells in each phase of interphase, mitosis (anaphase, metaphase and prophase) and cells demonstrating abnormal characteristics including cell elongation, blebbing, apoptotic bodies. Further quantitative data were supplied by means of cell number determination and flow cytometry. Three independent experiments were conducted where the mean and the standard deviation were calculated. Means were illustrated by bar charts and standard deviations were shown with errors bars. A *P*-value < 0.05 calculated by means of the Student *t*-test was used for statistically significance and was indicated by an asterisk (*). Flow cytometry analysis involved at least 10,000 events and was repeated three times. Flow cytometry data were analyzed using Cyflogic version 1.2.1 software (Pertu Therho, Turko, Finland).

## Results

### Cellular proliferation

**Fig. 1:**
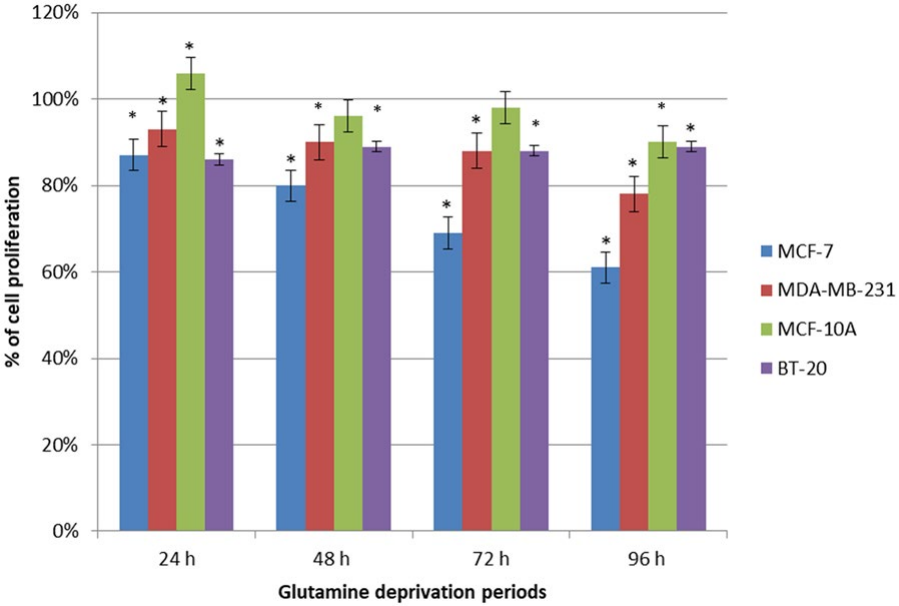
Glutamine deprivation results in time-dependent decreased cell growth. Graph illustrating cell growth after 24 h, 48 h, 72 h and 96 h of glutamine deprivation in MCF-7-, MDA-MB-231-, MCF-10A- and BT-20 cell lines. The MCF-7 cell line was most prominently affected. An asterisk indicates significance with *P*-value < 0.05 when compared to cells propagated in complete medium

Spectrophotometry results of crystal violet staining indicated a time-dependent decrease in proliferation after glutamine deprivation in both tumorigenic cell lines (Fig. 1). MCF-7 cells showed 87%, 80%, 69%, 62% cell growth after 24 h, 48 h, 72 h and 96 h deprivation from glutamine. MDA-MB-231 cells revealed 93%, 90%, 88% and 78% cell growth after 24 h, 48 h, 72 h and 96 h deprivation from glutamine. MCF-10A cells showed 108%, 94%, 98% and 89% cell growth after 24 h, 48 h, 72 h and 96 h deprivation from glutamine. BT-20 cell growth decreased to 86% after 24 h of glutamine deprivation and remained unchanged thereafter. These results suggest tumorigenic cell lines are more prominently affected by glutamine starvation when compared to the non-tumorigenic cell line. Furthermore, the tumorigenic breast estrogen receptor positive luminal cell line (MCF-7) was more prominently affected by glutamine deprivation when compared to the tumorigenic breast estrogen receptor negative basal cell line (MDA-MB-231).

### Morphology

**Fig. 2:**
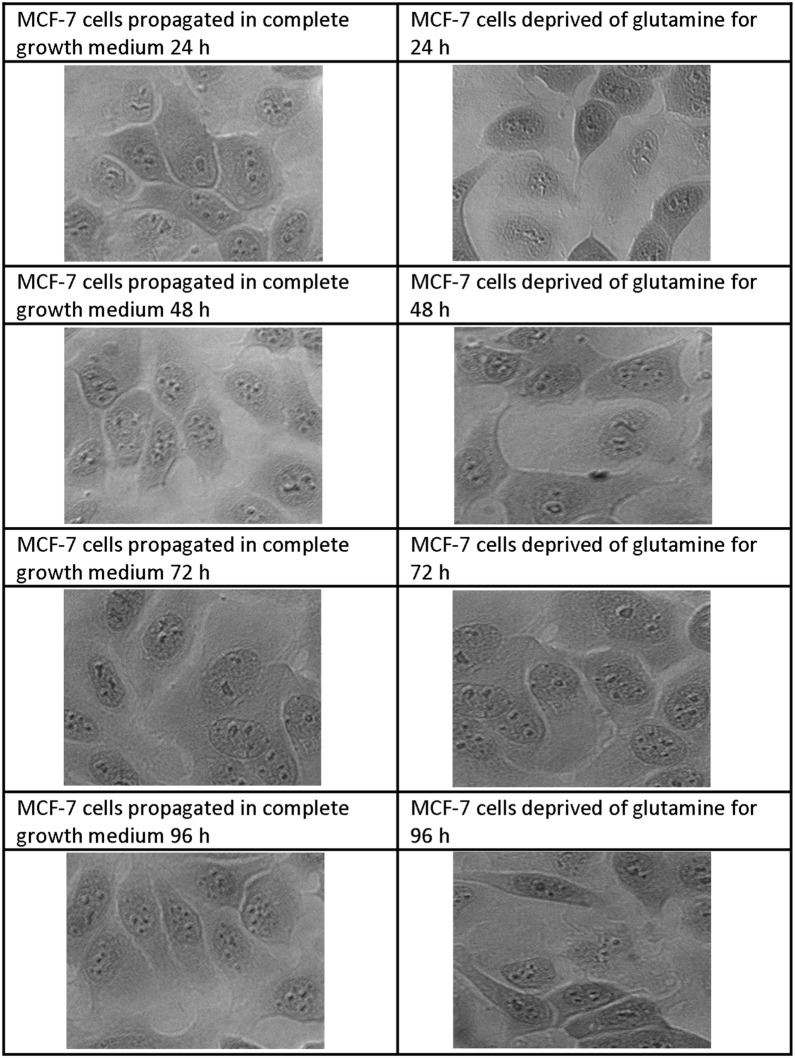
Light microscopy images demonstrating morphology of MCF-7 cells after glutamine deprivation. Morphology of the MCF-7 cell line after 24 h, 48 h, 72 h and 96 h of glutamine deprivation compared to MCF-7 cells propagated in complete growth medium 24 h, 48 h, 72 h and 96 h. Starvation of glutamine for (48 h, 72 h and 96 h) resulted in minor decreased cell density and the appearance of elongated cells when compared to cells propagated in growth medium (×20 magnification)

**Fig. 3:**
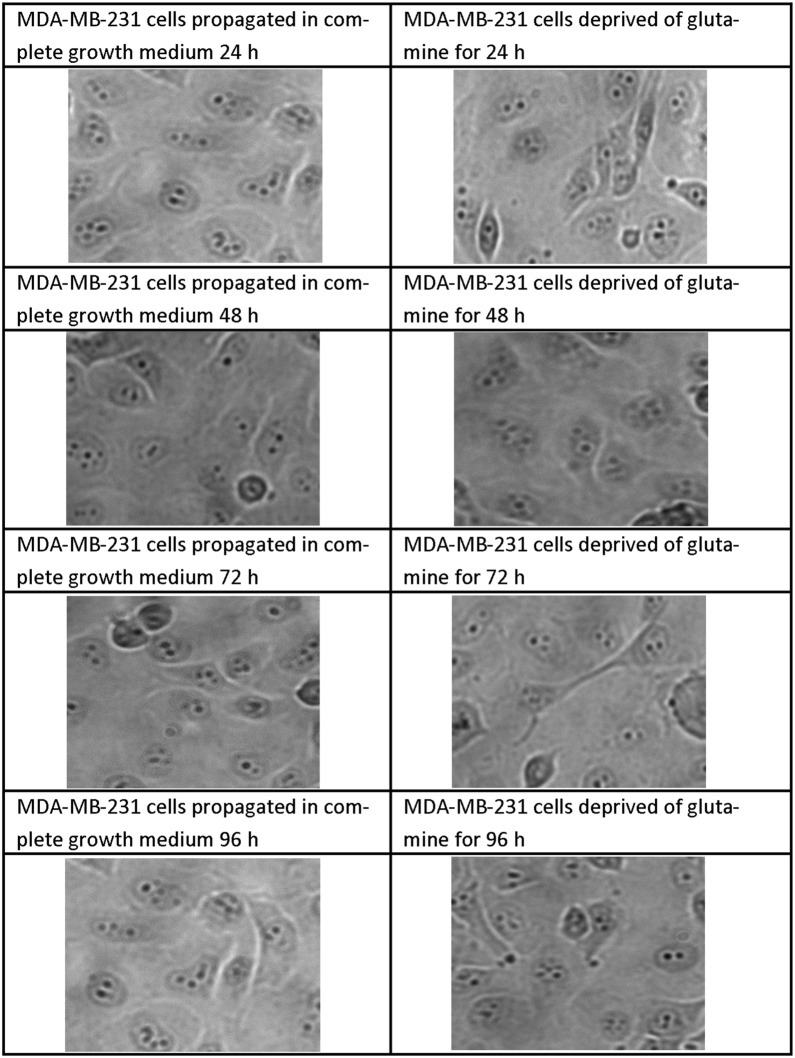
Light microscopy images demonstrating morphology of MDA-MB-231 cells after glutamine deprivation. Morphology of the MDA-MB-231 cell line after 24 h, 48 h, 72 h and 96 h of glutamine deprivation compared to MDA-MB-231 cells propagated in complete growth medium 24 h, 48 h, 72 h and 96 h. Glutamine deprivation for 96 h decreased cell density slightly when compared to cells propagated in complete growth medium. No other significant changes in morphology were observed after glutamine deprivation when compared to cells propagated in complete growth medium (×20 magnification)

**Fig. 4:**
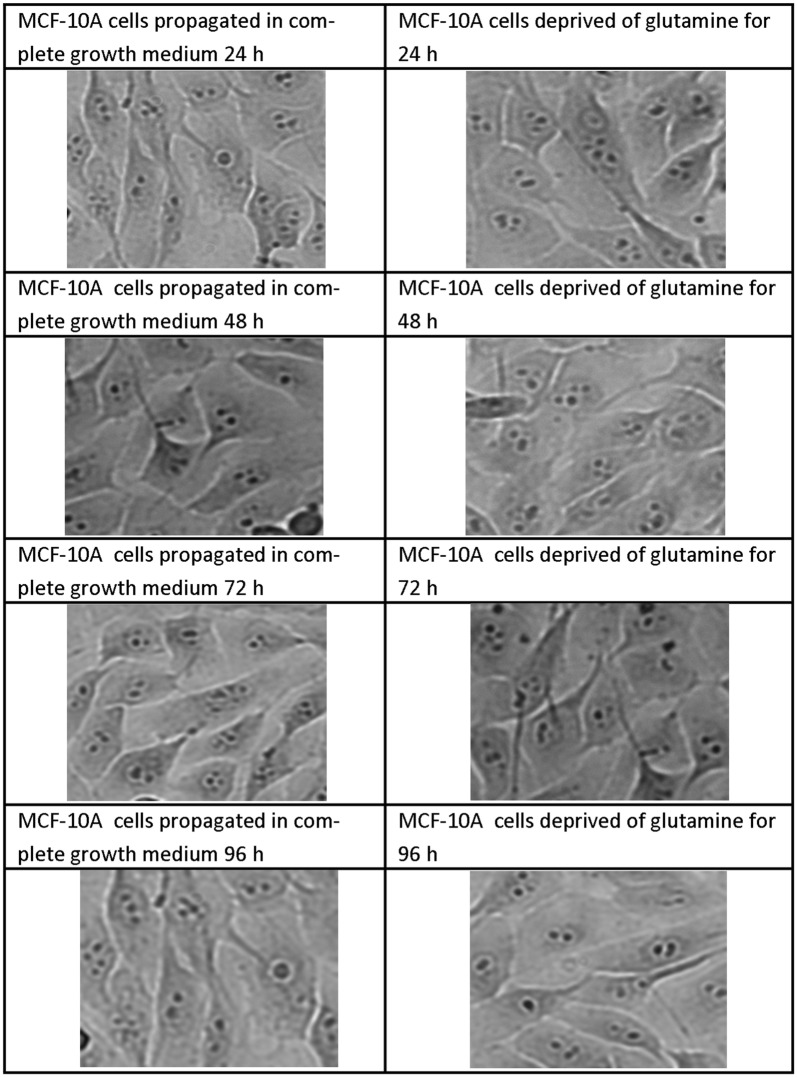
Light microscopy images demonstrating morphology of MCF-10A cells after glutamine deprivation. Morphology of the MCF-10A cell line after 24 h, 48 h, 72 h and 96 h of glutamine deprivation compared to MCF-10A cells propagated in complete growth medium 24 h, 48 h, 72 h and 96 h. Glutamine deprivation for 72 h and 96 h decreased cell density slightly when compared to cells propagated in complete growth medium. Furthermore, MCF-10A cells appeared rounded following glutamine deprivation when compared to cells propagated in complete growth medium (×20 magnification)

**Fig. 5:**
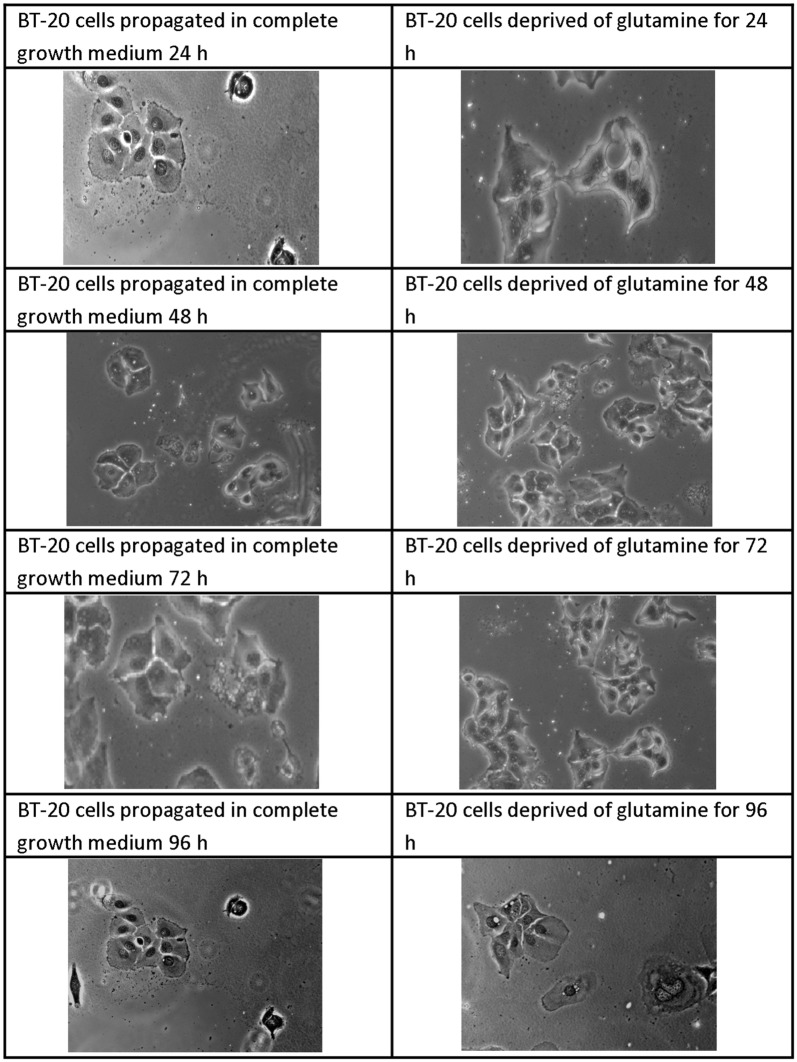
Light microscopy images demonstrating morphology of BT-20 cells after glutamine deprivation. Morphology of the BT-20 cell line after 24 h, 48 h, 72 h and 96 h of glutamine deprivation compared to BT-20 cells propagated in complete growth medium 24 h, 48 h, 72 h and 96 h. Glutamine deprivation for decreased cell density slightly when compared to cells propagated in complete growth medium. The BT-20 cell line displayed minimal effects on morphology due to glutamine deprivation (×20 magnification)

Light microscopy was used to visualize effects of glutamine deprivation on morphology by the use of haematoxylin and eosin stained samples (Figs. 2, 3, 4, 5). Glutamine deprivation resulted in compromised cell density in all the cell lines. Moreover, glutamine deprivation in the MCF-7 cells resulted in elongated cells. Glutamine deprivation resulted in rounded cells in the MCF-10A cell line. No statistically significant changes were observed in the mitotic indices (Additional file 1: Table S1).

### Oxidative stress

**Fig. 6:**
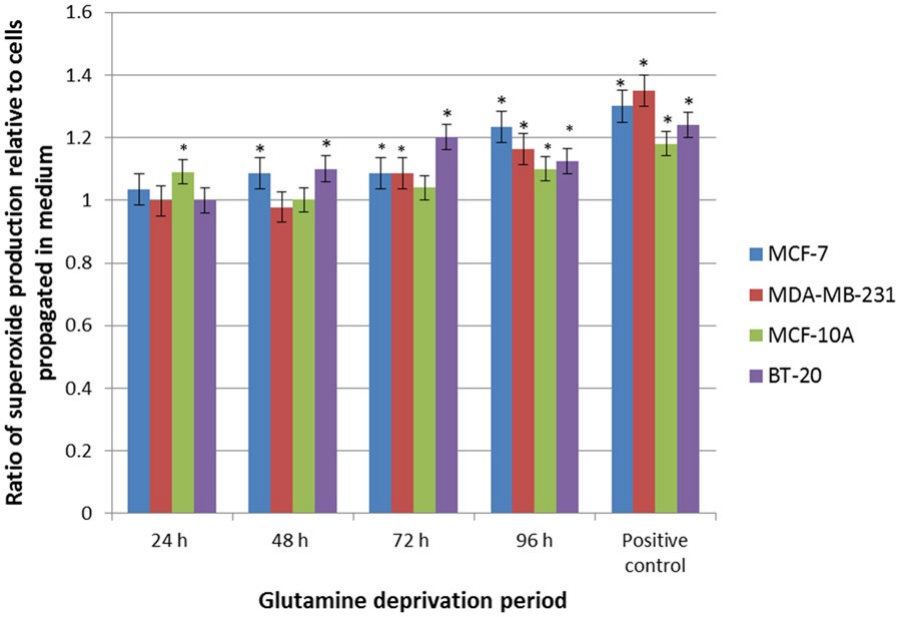
Glutamine deprivation results in increased superoxide production. Graph illustrating superoxide production after 24 h, 48 h and 72 h and 96 h of glutamine deprivation in MCF-7-, MDA-MB-231-, MCF-10A and BT-20 cell line. MCF-7-, MDA-MB-231 and BT-20 cell line illustrated elevated levels of superoxide production. MCF-10A illustrated a biphasic production of superoxide. An asterisk indicates significance with *P*-value < 0.05 when compared to cells propagated in complete medium

Dehydroethidium was utilized to investigate effects of glutamine deprivation on superoxide production. The MCF-7 cell line data demonstrated a significant increase in superoxide production to 1.23-fold after 96 h of glutamine deprivation when compared to cells propagated in complete growth medium (Fig. 6). The MDA-MB-231 cell line demonstrated a statically significant increase to 0.98- and 1.63-fold after 48 h, and 96 h of glutamine deprivation respectively when compared to cells propagated in complete medium. In addition, the MDA-MB-231 cell line showed increased superoxide production to 1.2 folds after 96 h of glutamine deprivation. Data from the MCF-10A cell line demonstrated a statically significant increase to 1.23-fold increase after 96 h of glutamine deprivation when compared to cells propagated in complete medium. Glutamine deprivation refrained from significantly increasing or decreasing superoxide production in the non-tumourigenic MCF-10A cell line. Superoxide production in the BT-20 cell line increased significantly in a time-dependent manner up to 1.20-fold increase after 72 h of deprivation from glutamine when compared to growth medium. Furthermore, superoxide production decreased thereafter despite continued glutamine deprivation till 96 h (Fig. 6).

**Fig. 7:**
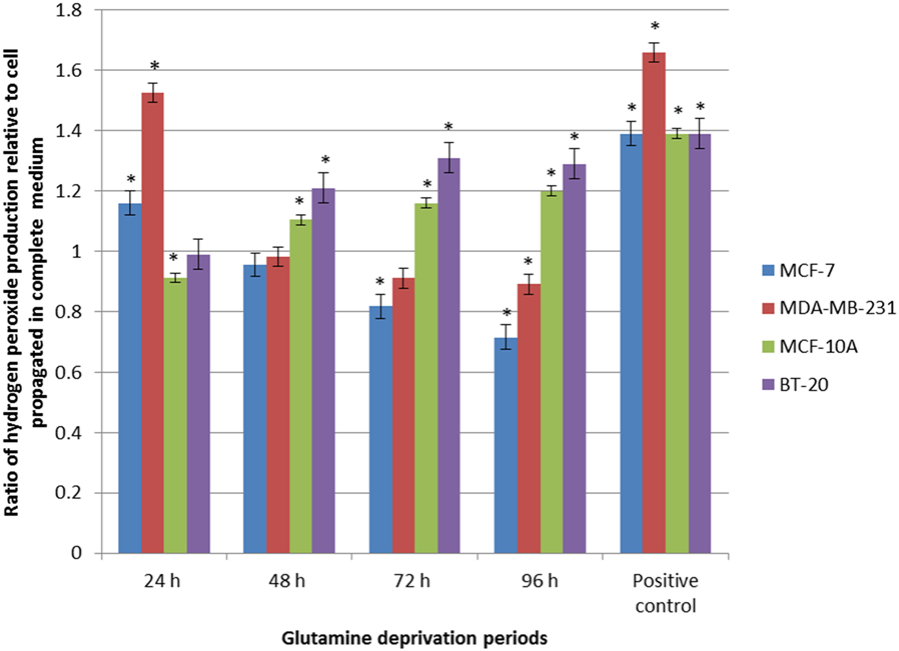
Glutamine deprivation results in aberrant hydrogen peroxide production. Graph illustrating hydrogen peroxide production after 24 h, 48 h, 72 h and 96 h of glutamine deprivation in MCF-7-, MDA-MB-231-, MCF-10A and BT-20 cell lines. The MCF-7 and MCF-10A cell lines demonstrated a temporal production of hydrogen peroxide whilst the MDA-MB-231 illustrated decreased levels of hydrogen peroxide. The MDA-MB-231 cell line was most prominently affected with generation of hydrogen peroxide rising to 1.53-fold when compared to cells propagated in complete growth medium. Levels of hydrogen peroxide decreased thereafter in a time-dependent manner to 0.89-fold after 96 h deprivation from glutamine relative to cells propagated in complete growth medium. The BT-20 cell line illustrated elevated levels of hydrogen peroxide levels relative to cells propagated in complete growth medium. An asterisk indicates significance with *P*-value < 0.05 when compared to cells propagated in complete medium

2, 7-Dichlorofluoresceindiacetate was utilized to investigate effects of glutamine deprivation on hydrogen peroxide production. Data demonstrated that glutamine deprivation in the MCF-7 cell line resulted in a significant increase in hydrogen peroxide production of 1.16-fold after 24 h of glutamine deprivation (Fig. 7). However, after 72 h and 96 h of glutamine deprivation, hydrogen peroxide production decreased to 0.82- and 0.72-fold respectively relative to cells propagated in complete growth medium. Glutamine deprivation in the MDA-MB-231 cell line resulted in a statically significant decrease of hydrogen peroxide production to 0.89-fold after 96 h relative to cells propagated in complete growth medium. However, 24 h deprivation of glutamine in the MDA-MB-231 cell line demonstrated a significant increase in hydrogen peroxide production to 1.53-fold when compared to cells propagated in complete growth medium. MCF-10A cell line data resulted in a time-dependent fold increase of 0.91 and 1.10 in hydrogen production after 24 h and 48 h of glutamine deprivation. A statistically significant fold increase of 1.29 was observed after 96 h of glutamine deprivation relative to cells propagated in complete growth medium. Hydrogen peroxide generation in the BT-20 cell line resulted in a statically significant increase of 1.21-, 1.31- and 1.29-fold after 48 h, 72 h and 96 h of glutamine deprivation. The BT-20 cell line illustrated a time-dependent increased production of hydrogen peroxide to 1.31 fold increase after 72 h deprivation from glutamine when compared to cells propagated ion complete growth medium (Fig. 7).

### Mitochondrial membrane potential

Mitochondria are essential for energy metabolism, redox regulation, calcium homeostasis and various other cellular functions. Mitochondria disruption is potentially fatal for the cell due to energy depletion, calcium dysregulation and oxidative stress [29]. The effect of glutamine deprivation on the mitochondrial membrane potential was evaluated as an indicator of cell health and injury.

**Fig. 8:**
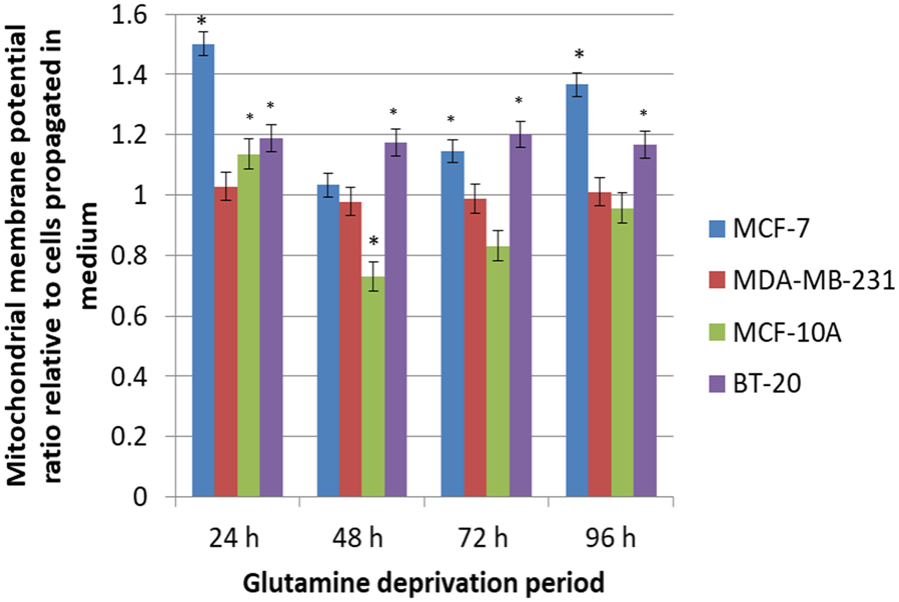
Glutamine deprivation results in compromised mitochondrial membrane potential. Graph illustrating mitochondrial membrane potential production after 24 h, 48 h, 72 h and 96 h of glutamine deprivation in MCF-7-, MDA-MB-231-, MCF-10A and BT-20 cell lines. MCF-7 and BT-20 cell line illustrated hyperpolarisation whilst the MDA-MB-231 and MCF-10A illustrated time-dependent, biphasic hyperpolarisation of the mitochondrial membrane potential. An asterisk indicates significance with *P*-value < 0.05 when compared to cells propagated in complete growth medium

The Mitocapture assay contains a cationic dye that accumulates in the mitochondria and fluoresces red in cells containing a hyperpolarised (negative) mitochondrial membrane. The cationic dye in a cell possessing a depolarised mitochondrial membrane will fluoresce green in the cytoplasm [30]. Thus, comparing cells cell deprived of glutamine with cells propagated in completed media, a fold increase above 1 is indicative of cells possessing a depolarised mitochondrial membrane potential. A fold decrease below 1 refers to cells possessing a hyperpolarized mitochondrial membrane potential. The MCF-7 cell line demonstrated a significant depolarisation after 24 h and 96 h (1.5- and 1.37-fold increase respectively) after deprivation from glutamine when compared to cells propagated in complete growth medium (Fig. 8). The mitochondrial membrane potential of the MDA-MB-231- and MCF-10A-cell lines remained unchanged over 96 h of glutamine deprivation. The mitochondrial membrane potential of the MCF-10A was depolarised after 24 h of glutamine deprivation in comparison with 48 h, 72 h and 96 h of glutamine deprivation which remained unchanged. The mitochondrial membrane potential of the BT-20 cell line was depolarised after 72 h of glutamine deprivation reaching 1.20-fold increase when compared to cells propagated in complete growth medium suggesting pro-death mechanisms; however, it remained unchanged at the other time intervals investigated (Fig. 8).

### Cell cycle progression

**Fig. 9:**
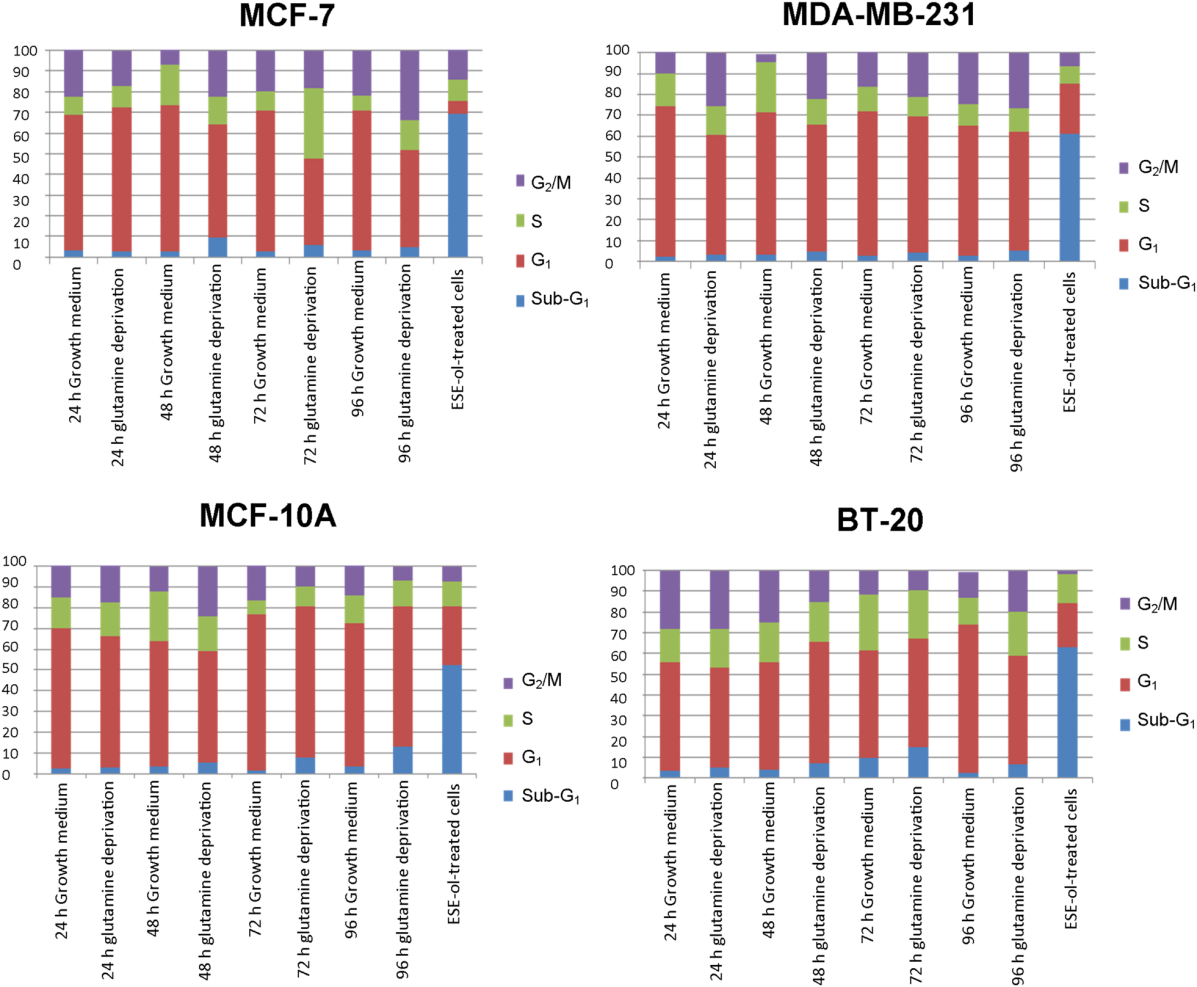
Glutamine deprivation results in aberrant changes in cell cycle progression. Graphs illustrating cell cycle progression after 24 h, 48 h, 72 h and 96 h of glutamine deprivation in MCF-7-, MDA-MB-231-, MCF-10A and BT-20 cell lines. MCF-7 cell line (top left) illustrated increased amount of cells in the S-phase. MDA-MB-231 cell line (top right) illustrated increased amount of cells present in the G_2_/M phase. MCF10-A cell line (bottom left) illustrated decreased amount of cells present in the G_1_ phase and BT-20 cell line (bottom right) illustrated increased cells present in the G_1_ phase. Additional file 1: Table S2 illustrate different phases of the cell cycle with the percentage of cells found in the particular phase where an asterisk indicates significance with *P*-value < 0.05 when compared to cells propagated in complete growth medium

The effects of glutamine deprivation on the cell cycle were investigated by means of flow cytometry. PI stains the DNA and thus enables the quantification of DNA correlating with stages of the cell cycle during cell division (Fig. 9 and Additional file 1). Glutamine deprivation for 72 h in the MCF-7 cell line demonstrated a significant decrease in the number of cells occupying the G_1_ phase (41%) accompanied by a significant increase in the number of cells occupying the S-phase (34%) when compared to cells propagated in complete growth medium (68%, 9.22% respectively) (Fig. 9). After 96 h of glutamine deprivation, the MCF-7 cell line demonstrated a significant decrease in the number of cells occupying the G_1_ phase (47%) accompanied by a significant increase in the number of cells occupying the G_2_/M phase (33%) when compared to cells propagated in complete medium (67.81%, 22% respectively). The MDA-MB-231 cell line resulted in a significant decrease in the number of cells present in the G_1_ phase after 24 h (25.51%), 48 h (61%) and 96 h (56%) of glutamine deprivation when compared to cells propagated in complete growth medium where 72%, 68% and 62% were observed. In addition, the MDA-MB-231 cell line illustrated a significant decrease in the number of cells occupying the S-phase after 48 h (12%) of glutamine deprivation when compared to the cells propagated in complete growth medium (24%). Glutamine deprivation of 24 h and 72 h displayed a significant increase of cells occupying the G_2_/M phase with 26% and 21% respectively when compared to cells propagated in complete growth medium where the amount of cells were 10% and 15% respectively. The MCF10-A cell line demonstrated a significant decrease in the cells present in the G_2_/M phase (10%) after 72 h of glutamine deprivation when compared to the cells propagated in complete growth medium (18%). The BT-20 cell line revealed a significant increase of cells occupying the G_1_ phase with 58% and 73% of cells present after 48 h and 72 h of glutamine deprivation when compared to cells propagated in complete growth medium with 47% and 52% respectively. However, after 96 h of glutamine deprivation 51% of cells occupied the G_1_ phase demonstrating a significant decrease when compared to cell propagated in complete growth medium (72%) (Fig. 9).

### Apoptosis induction

**Fig. 10:**
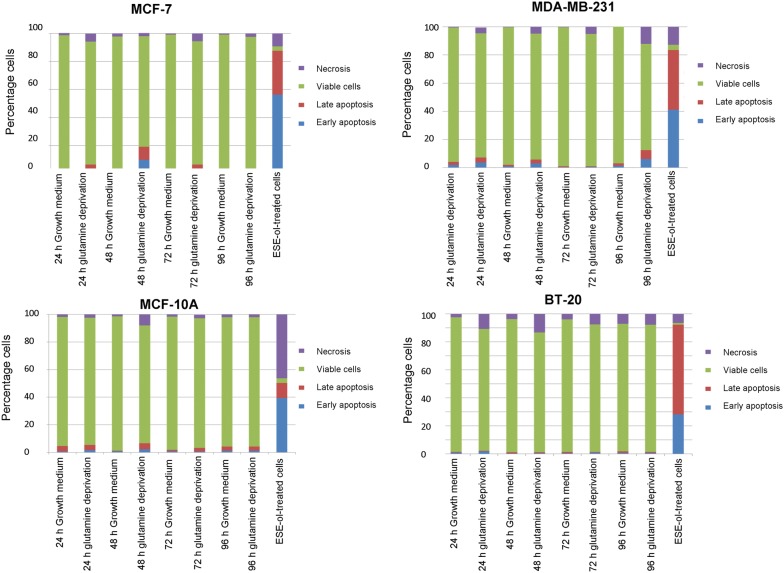
Glutamine deprivation results in the induction of apoptosis and necrosis. Graphs illustrating apoptosis induction after 24 h, 48 h, 72 h and 96 h of glutamine deprivation in MCF-7- (top left), MDA-MB-231- (top right), MCF-10A (bottom left) and BT-20 (bottom right) cell lines. Decreased cell viability was the most prominently observed following glutamine deprivation in the MCF-7 cell line (87.61% and 78.91%) after 24 h and 48 h. Viability in the MDA-MB-231 cell line decreased to 76% after 96 h of glutamine deprivation. The MCF10A- and BT-20 cell lines illustrated decreased cell viability to 94% and 91% respectively after 96 h of glutamine deprivation

The influence of glutamine deprivation on apoptosis and necrosis were quantified using flow cytometry in combination with Annexin V-FITC and PI. The MCF-7 cell line showed a significant decrease in the number of viable cells (87.61% and 78.91%) after 24 h and 48 h of glutamine deprivation when compared to cells propagated in complete growth medium (Fig. 6 and Additional file 1). The MDA-MB-231 cell line demonstrated a significant decrease in cell viability after 96 h of glutamine deprivation to 76% when compared to cells propagated in complete growth medium which presented with 97% of viable cells. The MCF10A- and BT-20 cell lines illustrated decreased cell viability to 94% and 91% respectively after 96 h of glutamine deprivation; however, no significant induction of apoptosis was observed (Fig. 10).

## Discussion

Although tumorigenic- and non-tumorigenic cells are both reliant on glutamine for survival, tumorigenic cells have a significantly elevated consumption rate of glutamine due to the significantly upregulated metabolic processes required for antioxidant activity, synthesis of macromolecules and maintaining the pool of intermediates in metabolic pathways [31]. Due to these upregulated metabolic activities, tumorigenic cells utilize higher quantities of glutamine and glucose when compared to non-tumorigenic and senescent cells [32]. This is supported by studies that demonstrated that several types of cancer is characterised by glutamine metabolism dysfunction and glutamine addiction compared to non-tumourigenic tissue- and cells [33]. Thus, tumorigenic cells modify essential metabolic pathways (glycolysis, pentose phosphate pathway and glutaminolysis) and alter cancer-related gene expression (myelocytomatosis oncogene, p53 and pyruvate kinase M2) [34]. Recently, there is renewed interest in studying the importance of glutamine and other nutrients essential for tumour growth and cell survival since improved understanding regarding the crosstalk between glutamine, proliferation and evasion of cell death could be exploited in glycolysis or glutaminolysis for improved therapeutic purposes [3]. In order to fully take advantage of tumorigenic aberrant metabolism for potential future therapeutics it is necessary to comprehend how glutamine is involved in tumorigenic proliferation, redox regulation, cell survival and cell death. In this study, the influence of glutamine deprivation on cellular proliferation, morphology, ROS production, mitochondrial membrane potential and apoptosis induction were investigated in tumorigenic- and non-tumorigenic breast cell lines.

Spectrophotometry data indicated that glutamine deprivation resulted in time-dependent antiproliferative effects in MCF-7- and MDA-MB-231 cell lines. In addition, our study demonstrated that glutamine deprivation resulted in more prominent antiproliferative activity in the luminal MCF-7 cell line when compared to the basal MDA-MB-231-, MCF-10A- and BT-20 cell lines. This suggests that the estrogen tumorigenic estrogen receptor positive luminal cell line is more dependent on glutamine as an energy source when compared to basal triple negative cell line BT-20- and MDA-MB-231 cell lines suggesting that tumorigenic- and metastatic cells are dependent on glutamine for proliferation. The resistance may be a result of the cell type’s ability to synthesize de novo glutamine or opt for alternative routes for an anaplerotic substrate to provide alternative energy producing pathways where carbon and nitrogen sources are delivered to the Krebs cycle via alpha-ketoglutarate. The latter then leads to further supply and support the cell with adequate energy which allows these cell lines to survive following glutamine deprivation [35]. Previous studies done via MTT assays have also reported that glutamine deprivation for 48 h resulted in a dose-dependent decrease in cell growth and metabolism in breast cell lines including human breast metastatic epithelial ductal carcinoma cell line (T47D), human breast non-metastatic ductal carcinoma cell line (BT474), MCF-7, human breast metastatic epithelial adenocarcinoma cell line (MDA-MB-361), BT-20, MDA-MB-231 and human breast epithelial medullary carcinoma cell line (MDA-MB-157) [24]. Furthermore, the current study demonstrated that glutamine deprivation least affected the non-tumorigenic MCF-10A cell line. This indicates that tumorigenic cell lines are more susceptible to glutamine deprivation when compared to non-tumorigenic cells. In addition, these results confirm that glutamine is a critical nutrient and promotes optimal cell proliferation.

Superoxide is an anion radical and is a precursor of a variety of reactive free radicals which will thus constitute to being ROS [36]. The production of superoxide in the MCF-7 cell line was increased after 96 h of glutamine deprivation and was most prominently affected when compared to the MDA-MB-231-, MCF-10A and BT-20 cell lines. These results were confirmed by Aykin-Burns et al. [37], where breast cancer cell lines illustrated elevated levels of superoxide. Increased metabolism and subsequent elevated superoxide production will lead to increased levels of various different ROS [38].

Flow cytometry data demonstrated that ROS (hydrogen peroxide) production after glutamine deprivation is time-dependent. Hydrogen peroxide production was most prominently affected in the MDA-MB-231 after 24 h glutamine deprivation. This finding was also confirmed by another study where ROS was increased in the ovarian cancer cell lines HEY-, SKOV3- and IGOROV-1 after 24 h of glutamine deprivation [39]. Hydrogen peroxide with concentrations above 100 µM is known to be cytotoxic and induces DNA damage and thus inhibits proliferation [40]. The decreased cell proliferation studies after 24 h of glutamine deprivation in the MCF-7 cell line correlate with increased hydrogen peroxide production and also confirm that significantly elevated ROS levels are detrimental to cell growth. This result was also observed by Vilema-Eriquez et al. [40] where hydrogen peroxide production (50–200 µM) inhibited proliferation in the MCF-7 cell line. However, hydrogen peroxide generation decreased thereafter in a time-dependent manner in both the MDA-MB-231 and MCF-7 tumorigenic cell lines. High ROS concentrations are damaging to lipids, proteins and DNA. Ample evidence exists demonstrating that low and intermediary levels of ROS have physiological importance which include cellular responses to noxia, defence against infectious agents, participation in cellular signalling pathways and induction of mitogenic responses [41]. The latter is supported by our results, where the BT-20 cell line demonstrated a statistically significant increase in the hydrogen peroxide production in a time-dependent manner following glutamine deprivation which correlated with the unchanged cell growth observed in this cell line. Hydrogen peroxide results from antioxidant defense mechanisms where superoxide is neutralized by superoxide dismutase which reduces superoxide anions to hydrogen peroxide which is then further detoxified by catalase into water and oxygen thus increased levels of hydrogen peroxide levels can also be resultant of upregulated superoxide dismutase production [42]. The increased- and decreased quantities of ROS observed in this study following glutamine deprivation are time- and cell line-dependent and is possibly due to the subsequent shortage of carbon sources that supplies the Krebs cycle with intermediates to further support the survival of tumourigenic cells indicating a possible shift in cellular metabolism from glutaminolysis to alternative anaplerotic pathways that support pyruvate carboxylation [43]. The shift in metabolism is supported by the spectrometry results of the BT-20 results, where, after 24 h of glutamine deprivation, proliferation was stabilized which are also seen in the production of hydrogen peroxide where, after 24 h of glutamine deprivation, levels of hydrogen peroxide were increased.

Analysis of the mitochondrial membrane potential using flow cytometry resulted in the hyperpolarisation of the mitochondrial membrane potential of the MCF-7- and BT-20 cell lines and time-dependent hyperpolarisation in the MDA-MB-231- and MCF-10A cell lines. Eukaryotic cells must maintain a hyperpolarised mitochondrial membrane potential in order to avoid the release of pro-apoptotic agents such as *cytochrome c* which will subsequently induce apoptosis. However, tumorigenic cells have elevated levels of hyperpolarisation as a prosurvival mechanism as described by Forrest et al. [44]. Sastre-Serra et al. [45], suggests that an increase with mitochondrial membrane potential which is termed hyperpolarisation, enhances ROS production and thus promote tumorigenicity. Figures 2 and 3 indicated hyperpolarisation which correlated with increased ROS production. Furthermore, the BT-20 cell line showed no antiproliferative effects further confirming adaptation following glutamine deprivation. Dmitry et al. [46], reported that hyperpolarisation of the mitochondrial membrane potential coincided with the generation of excessive ROS (hydrogen peroxide) production, which further reiterates the findings of this research paper. The presence of transient hyperpolarisation is observed in the MDA-MB-231 cell line and may possibly be explained by a temporal blockage in the electron transfer chain [45]. Thus, mitochondria are able to withstand high levels of oxidative stress and develop adaptive mechanism to ensure optimal functioning. Furthermore, this adaptation supports tumorigenicity [44].

Cell cycle progression analysis by flow cytometry illustrated an increase in the number of cells present in the S-phase in the MCF-7 cell line after 72 h of glutamine deprivation accompanied by a significant decrease in the G_1_ phase of the cell cycle. This suggests nucleotide depletion, obtained through chemical inhibition of biosynthesis that may induce slower cycling cells in the S-phase as elucidated by Gaglio et al. [47] due to inadequate pairing of nucleotide bases. Cyclin D1 and cyclin-dependent kinase 4 which promote the entry of cells into the S-phase are upregulated by glutamine in a concentration-dependent manner. The MDA-MB-231 cell line resulted in a significant increase in the G_2_/M phase after 24 h of glutamine deprivation which was also found by Kansara et al. [48] who demonstrated that the fibrosarcoma cell line, KHT-C2-LP1, showed a significant number of cells present in the G_2_/M phase of the cell cycle suggesting there is rapid protein synthesis due to the cells efforts to adapt to the lack of glutamine and thus adequate carbon sources needed for sufficient energy production. MCF10-A cells demonstrated a significant decrease in the G_1_ phase after 96 h of glutamine deprivation. Glutamine deprivation in the BT-20 cell line resulted in a significant increase G_1_ phase after 72 h of glutamine deprivation. These results suggest that glutamine deprivation differentially affects cell cycle progression phases in a time-dependent manner.

Annexin V paired with flow cytometry was utilized to detect the phosphatidylserine flip which demonstrated that the MCF-7 cell line is the only cell line to be significantly affected with regards to apoptosis induction after glutamine deprivation. These studies demonstrated that glutamine deprivation reduced cell viability in a time-dependent manner in the MCF-7 cell line to 75% after 96 h with a corresponding increase in cells observed in apoptosis and necrosis when compared to cells propagated in complete growth medium. This corresponds with earlier findings from the present study where glutamine deprivation resulted in decreased cell proliferation, oxidative stress and disruption in the mitochondrial membrane potential in the MCF-7 cell line indicating the induction of apoptosis by means of the mitochondrial pathway.

## Conclusion

Upregulated glutamine consumption required for sustainable energy and macromolecule production in cancer cells may result in specific metabolic vulnerabilities that can possibly targeted for therapy. However, it still remains to be elucidated what type of tumors and what molecular characteristics can be associated with a favorable outcome [49]. This study found that glutamine deprivation results in differential antiproliferative- and aberrant redox activity, however, negligible apoptosis induction was observed. All of the above-mentioned effects were more prominently observed in the MCF-7 cell line when compared to the MDA-MB-231 cell line. The non-tumourigenic MCF-10A and non-metastatic BT-20 cell were affected the least by glutamine deprivation. This study provides evidence that there are differential responses in different types of tumourigenic breast cell lines while leaving non-tumourigenic cells less affected. Understanding how cells adapt to glutamine deprivation is essential since more therapeutics are being developed that target cancer cell metabolism. Furthermore, data gained from glutamine deficiency studies may lead to translational possibilities to improve the manner in which we diagnose, monitor, and treat cancer. Future studies will involve investigating the possibility that glutamine deprivation potentially possess synergic effects in combination with current chemotherapeutic strategies.

## Additional file

**Additional file 1.** Table of mitotic indices, table of cell cycle progression and table of Annexin V/PI staining.

## Abbreviations

DCFDA: 2, 7-dichlorofluoresceindiacetate
ATP: adenosine triphosphate
DHE: dehydroethidium
DMEM: Dulbecco’s minimum essential medium eagle
ER: estrogen receptor
FITC: fluorescein isothiocyanate
HER2: human epidermal growth factor receptor 2
PBS: phosphate-buffered saline
PR: progesterone receptor
PI: propidium iodide
ROS: reactive oxygen species
TRAIL: tumour necrosis factor related apoptosis inducing ligand
