# Supplementary material for: Effects of glutamine deprivation on oxidative stress and cell survival in breast cell lines
Source: Biol Res. 2019 Mar 27;52:15. doi: 10.1186/s40659-019-0224-9 (PMC6437944; doi:10.1186/s40659-019-0224-9)
Supplement: Supplementary file 1 — Additional file 1. Table of mitotic indices, table of cell cycle progression and table of Annexin V/PI staining. [file 40659_2019_224_MOESM1_ESM.doc]

**Additional data**

Table S1: Mitotic indices of MCF-7-, MDA-MB-231-, MCF-10A and BT-20 cell lines:

| **Condition** | **MCF-7** | **MDA-MB-231** | **MCF10-A** | **BT-20** |
| --- | --- | --- | --- | --- |
| **24 h Medium only** | **Average ± SD (%)** | **Average ± SD (%)** | **Average ± SD (%)** | **Average ± SD (%)** |
| Interphase | 96.62 ± 0.91 | 97.41 ± 1.35 | 95.92 ± 2.3 | 97.3± 2.78 |
| Prophase | 1.45 ± 0.21 | 1.38 ± 1.1 | 2.87 ± 0.36 | 1.24± 3.6 |
| Metaphase | 0.677 ± 0.34 | 0.85 ± 0.34 | 0.76 ± 0.27 | 0.69± 1.87 |
| Telophase | 0.23 ± 0.19 | 0.2 ± 0.15 | 0.09 ± 0.13 | 0.26± 1.01 |
| Anaphase | 0.49 ± 0.25 | 0.17 ± 0.6 | 0.14 ± 0.25 | 0.18± 2.69 |
| Apoptosis | 0.09 ± 0.13 | 0.00 | 0.11 ± 0.12 | 0.007± 0.74 |
| Abnormal | 0.44 ± 0.38 | 0.00 | 0.11± 4.39 | 0.014± 0.36 |
| **24 h without Glutamine** | **Average ± SD (%)** | **Average ± SD (%)** | **Average ± SD (%)** | **Average ± SD (%)** |
| Interphase | 94.97 ± 2.65 | 95.85 ± 1.67 | 97.36 ± 0.43 | 98.36 ± 0.37 |
| Prophase | 1.39 ± 0.43 | 0.75 ± 0.37 | 1.05 ± 1.96 | 0.72 ± 0.65 |
| Metaphase | 0.68 ± 0.29 | 1.06 ± 0.19 | 1.07 ± 2.36 | 0.63 ± 0.15 |
| Telophase | 0.22 ± 0.17 | 0.55 ± 0.23 | 0.19 ± 0.37 | 0.2 ± 0.58 |
| Anaphase | 0.45 ± 0.29 | 0.48 ± 0.13 | 0.26 ± 2.94 | 0.012 ± 0.18 |
| Apoptosis | 0.76 ± 0.6 | 0.00 | 0.09 ± 0.42 | 0.03 ± 0.47 |
| Abnormal | 1.53 ± 1.08 | 1.32 ± 1.86 | 0.025 ± 0.78 | 0.006 ± 0.63 |
| **48 h Medium only** | **Average ± SD (%)** | **Average ± SD (%)** | **Average ± SD (%)** | **Average ± SD (%)** |
| Interphase | 96.48 ± 1.08 | 95.63 ± 0.09 | 97.38 ± 1.26 | 95.63 ± 1.36 |
| Prophase | 1.89 ± 0.97 | 2.03 ± 2.36 | 1.09 ± 0.39 | 2.63 ± 1.43 |
| Metaphase | 0.97 ± 2.3 | 0.85 ± 0.08 | 0.75 ± 2.98 | 0.68 ± 1.68 |
| Telophase | 0.21 ± 0.02 | 0.36 ± 1.83 | 0.016 ± 1.47 | 0.28 ± 1.23 |
| Anaphase | 0.42 ± 2.36 | 0.6 ± 1.67 | 0.34 ± 1.74 | 0.08 ± 1.37 |
| Apoptosis | 0.0134 ± 4.3 | 0.02 ± 0.41 | 0.05 ± 1.8 | 0.135 ± 1.63 |
| Abnormal | 0.007 ± 1.36 | 0.51 ± 1.07 | 0.374 ± 0.88 | 0.565 ± 1.18 |

| **48 h without Glutamine** | **Average ± SD (%)** | **Average ± SD (%)** | **Average ± SD (%)** | **Average ± SD (%)** |
| --- | --- | --- | --- | --- |
| Interphase | 97.8 ± 0.52 | 97.08± 0.71 | 95.85 ± 1.67 | 96.36± 0.29 |
| Prophase | 0.91 ± 0.37 | 1.23 ± 0.07 | 1.23 ± 0.07 | 1.61± 0.87 |
| Metaphase | 0.27 ± 0.13 | 0.92 ± 0.09 | 0.39 ± 0.89 | 0.29± 0.5 |
| Telophase | 0.24 ± 0.2 | 0.21 ± 0.02 | 0.27 ± 2.43 | 0.87± 1.15 |
| Anaphase | 0.23 ± 0.13 | 0.21 ± 0.08 | 0.36± 1.91 | 0.8± 1.67 |
| Apoptosis | 0.41 ± 0.45 | 0.2 ± 0.14 | 1.8± 0.57 | 0.07± 0.46 |
| Abnormal | 0.86 ± 0.66 | 0.11 ± 0.09 | 0.1± 2.12 | 0.1± 1.89 |
| **72 h Medium only** | **Average ± SD (%)** | **Average ± SD (%)** | **Average ± SD (%)** | **Average ± SD (%)** |
| Interphase | 97.63± 0.78 | 96.97± 0.43 | 97.25± 0.69 | 96.86± 0.23 |
| Prophase | 1.24± 0.12 | 1.39± 0.50 | 0.87± 0.74 | 0.89± 0.29 |
| Metaphase | 0.18± 0.66 | 0.25± 1.97 | 0.21± 1.87 | 0.34± 0.263 |
| Telophase | 0.29± 2.39 | 0.32± 2.78 | 0.17± 5.3 | 0.21± 0.78 |
| Anaphase | 0.12± 1.37 | 0.19± 1.74 | 0.01± 1.75 | 0.016± 0.82 |
| Apoptosis | 0.15±1.69 | 0.2± 3.37 | 0.53± 4.12 | 0.58± 0.26 |
| Abnormal | 0.39± 0.87 | 0.68± 0.47 | 0.96± 1.68 | 1.1± 0.78 |
| **72 h without Glutamine** | **Average ± SD (%)** | **Average ± SD (%)** | **Average ± SD (%)** | **Average ± SD (%)** |
| Interphase | 97.6± 0.1 | 96.74± 0.63 | 95.3± 0.73 | 96.36± 0.27 |
| Prophase | 1.09± 1.23 | 1.348± 0.24 | 1.28± 0.53 | 1.36± 0.43 |
| Metaphase | 0.11± 3.9 | 0.26± 0.29 | 0.27± 0.13 | 0.11± 0.63 |
| Telophase | 0.01± 0.78 | 0.29± 0.07 | 0.31± 1.67 | 0.213± 0.42 |
| Anaphase | 0.00 | 0.2± 0.05 | 0.02± 3.98 | 0.26± 0.68 |
| Apoptosis | 0.19± 0.09 | 0.137± 0.42 | 0.36± 2.31 | 0.39± 0.73 |
| Abnormal | 1.03± 0.88 | 1.03± 0.23 | 1.23± 3.77 | 1.3± 1.7 |
| **96 h Medium only** | **Average ± SD (%)** | **Average ± SD (%)** | **Average ± SD (%)** | **Average ± SD (%)** |
| Interphase | 97.31± 0.56 | 96.41± 0.65 | 95.8± 0.63 | 94.96± 0.15 |
| Prophase | 1.22± 1.92 | 1.3± 0.72 | 1.28± 0.27 | 1.36± 0.97 |
| Metaphase | 1.01± 0.49 | 0.98± 0.73 | 0.64± 0.87 | 0.29± 0.56 |
| Telophase | 0.03± 1.6 | 0.15± 0.85 | 0.067± 1.69 | 0.14± 0.14 |
| Anaphase | 0.15± 4.36 | 0.036± 0.38 | 0.23± 2.37 | 0.27± 0.73 |
| Apoptosis | 0.12± 2.36 | 0.12± 0.974 | 0.97± 5.01 | 0.19± 1.17 |
| Abnormal | 0.16± 1.66 | 1.04± 0.15 | 1.01± 1.69 | 2.79± 1.98 |
| **96 h without Glutamine** | **Average ± SD (%)** | **Average ± SD (%)** | **Average ± SD (%)** | **Average ± SD (%)** |
| Interphase | 96.3± 1.67 | 97.96± 1.29 | 96.34± 0.85 | 95.6± 0.39 |
| Prophase | 1.36± 2.39 | 1.48± 2.97 | 1.02± 0.57 | 1.43± 0.45 |
| Metaphase | 0.23± 1.97 | 0.93± 2.35 | 0.89± 1.96 | 0.52± 0.89 |
| Telophase | 0.17± 0.56 | 0.236± 4.85 | 0.01± 2.39 | 0.019± 0.78 |
| Anaphase | 0.187± 0.42 | 0.013± 1.48 | 0.23± 3.97 | 0.028± 1.02 |
| Apoptosis | 0.75± 0.63 | 0.016± 3.55 | 0.41± 1.89 | 0.013± 3.09 |
| Abnormal | 1.26± 0.41 | 0.014± 1.99 | 1.1± 1.29 | 2.39± 1.023 |
| **ESE-ol treated 48h** | **Average ± SD (%)** | **Average ± SD (%)** | **Average ± SD (%)** | **Average ± SD (%)** |
| Interphase | 80.19 ± 1.46 | 92.81 ± 1.63 | 94.36± 1.84 | 95.47± 0.56 |
| Prophase | 0.51 ± 0.13 | 0.45 ± 0.33 | 0.56± 1.04 | 0.49± 0.26 |
| Metaphase | 0.44 ± 0.04 | 0.18 ± 0.13 | 0.47± 2.89 | 0.37± 1.35 |
| Telophase | 0.14 ± 0.16 | 0.00 | 0.014± 0.96 | 0.00 |
| Anaphase | 0.59 ± 0.43 | 0.09 ± 0.06 | 0.36± 0.41 | 0.53± 3.62 |
| Apoptosis | 9.35 ±0.72 | 2.57 ± 0.65 | 2.97± 1.3 | 2.98± 2.69 |
| Abnormal | 8.9 ± 0.52 | 4.12 ± 2.45 | 1.6± 3.62 | 0.16± 4.23 |

Table S2 Different phases of the cell cycle after glutamine deprivation.

| **Condition** | **MCF-7** | **MDA-MB-231** | **MCF10-A** | **BT-20** |
| --- | --- | --- | --- | --- |
| **24 h Medium only** | **Average ± SD (%)** | **Average ± SD (%)** | **Average ± SD (%)** | **Average ± SD (%)** |
| Sub G1 | 3.37 ± 1.54 | 2.32 ± 1.94 | 2.4 ± 1.79 | 3.40 ± 4.42 |
| G1 | 65.36 ± 1.97 | 72.13 ± 4.25 | 67.89 ± 4.76 | 52.29 ±3.69 |
| S | 8.97 ± 3.98 | 15.39 ± 1.52 | 14.52 ± 1.987 | 16.19 ± 4.83 |
| G2/M | 22.48 ± 4.98 | 10.35 ± 2.39 | 16.1 ± 3.85 | 29.08 ± 2.39 |
| **24 h without Glutamine** | **Average ± SD (%)** | **Average ± SD (%)** | **Average ± SD (%)** | **Average ± SD (%)** |
| Sub G1 | 2.36 ± 2.96 | 3.12 ± 0.394 | 2.95 ± 4.85 | 5.06 ± 0.21 |
| G1 | 69.69 ± 3.96 | 57.49 ± 3.99* | 63.16 ± 4.07 | 48.33 ± 3.69 |
| S | 10.47 ± 0.79 | 13.86 ± 2.96 | 15.33 ± 3.26 | 18.34 ± 4.83 |
| G2/M | 17.08 ± 2.28 | 25.51 ± 3.85* | 18.56 ±1.36 | 29.25 ± 0.63 |
| **48 h Medium only** | **Average ± SD (%)** | **Average ± SD (%)** | **Average ± SD (%)** | **Average ± SD (%)** |
| Sub G1 | 2.69 ± 4.69 | 3.23 ± 0.33 | 3.62 ± 4.25 | 3.89 ± 3.97 |
| G1 | 70.96 ± 2.39 | 68.39 ± 4.11 | 60.05 ± 4.6 | 47.05 ± 1.54 |
| S | 19.3 ± 1.44 | 23.6 ± 2.04 | 24.02 ± 5.01 | 15.087 ± 4.22 |
| G2/M | 8.03 ± 0.18 | 4.086 ± 2.01 | 12.38 ± 3.59 | 20.85 ± 4.23 |
| **48 h without Glutamine** | **Average ± SD (%)** | **Average ± SD (%)** | **Average ± SD (%)** | **Average ± SD (%)** |
| Sub G1 | 9.38 ± 1.87 | 4.84 ± 1.23 | 5.21 ± 1.17 | 7.29 ± 3.57 |
| G1 | 54.58 ± 2.92* | 60.72 ± 1.31* | 54.07 ± 4.03 | 58.38 ± 4.57* |
| S | 13.66 ± 1.99 | 12.00 ± 1.79* | 16.75 ± 1.96 | 19.31 ± 1.61 |
| G2/M | 22.36 ± 4.69* | 22.41 ± 3.83 | 23.95 ± 2.28 | 15 ± 4.28 |
| **72 h Medium only** | **Average ± SD (%)** | **Average ± SD (%)** | **Average ± SD (%)** | **Average ± SD (%)** |
| Sub G1 | 2.73 ± 0.90 | 2.69 ± 3.24 | 1.77 ± 1.19 | 3.56 ± 1.28 |
| G1 | 68.26 ± 2.78 | 69.05 ± 1.19 | 75.25 ± 4.03 | 51.82 ± 2.97 |
| S | 9.22 ± 1.96 | 12.10 ± 2.27 | 6.76 ± 1.58 | 20.95 ± 2.4 |
| G2/M | 19.78 ± 3.82 | 14.51 ± 3.16 | 17.22 ± 1.87 | 12.54 ± 3.97 |
| **72 h without Glutamine** | **Average ± SD (%)** | **Average ± SD (%)** | **Average ± SD (%)** | **Average ± SD (%)** |
| Sub G1 | 6.08 ± 3.95 | 4.40 ± 2.19 | 7.78 ± 1.57 | 1.46 ±0.33 |
| G1 | 41.33 ± 2.22* | 65.01 ± 3.97 | 72.76 ± 4.26 | 73.4 ± 1.19* |
| S | 34.38 ± 4.87* | 9.53 ± 1.57 | 9.54 ± 0.53 | 14.42 ± 4.23 |
| G2/M | 18.19 ± 2.36 | 21.05 ± 4.36* | 9.91 ± 4.82* | 11.54 ± 4.46 |
| **96 h Medium only** | **Average ± SD (%)** | **Average ± SD (%)** | **Average ± SD (%)** | **Average ± SD (%)** |
| Sub G1 | 3.18 ± 0.86 | 2.56 ± 1.23 | 3.42 ± 1.11 | 2.26 ±0.62 |
| G1 | 67.81 ± 1.82 | 62.48 ± 1.31 | 69.04 ± 2.61 | 71.56 ± 1.75 |
| S | 7.09 ± 1.21 | 10.46 ± 1.79 | 13.44 ±0.79 | 12.96 ± 0.91 |
| G2/M | 21.9 ± 3.18 | 24.49 ± 3.83 | 14.37 ± 4.18 | 12.54 ±3.24 |
| **96 h without Glutamine** | **Average ± SD (%)** | **Average ± SD (%)** | **Average ± SD (%)** | **Average ± SD (%)** |
| Sub G1 | 4.89 ± 1.04 | 5.38 ± 0.85 | 12.93 ± 1.73 | 4.95 ± 0.86 |
| G1 | 46.89 ± 3.061* | 56.47 ± 1.66* | 67.79 ± 4.76 | 51.39 ± 1.62* |
| S | 14.66 ±1.85 | 11.75 ± 2.03 | 12.64 ± 1.42 | 18.42 ± 0.54 |
| G2/M | 33.54 ± 4.32* | 26.39 ± 1.90 | 6.64 ± 3.39* | 19.41 ± 2.05 |
| **ESE-ol treated 48h** | **Average ± SD (%)** | **Average ± SD (%)** | **Average ± SD (%)** | **Average ± SD (%)** |
| Sub G1 | 69.3 ± 4.36 * | 61.3 ± 2.42* | 52.36 ± 3.32* | 63.203 ±4.36* |
| G1 | 6.3 ± 2.97 | 23.6 ± 3.95 | 28.3 ± 3.85 | 21.2046 ± 3.24 |
| S | 10.39 ±1.92 | 8.64 ± 4.91 | 11.78 ± 1.36 | 13.7893 ± 4.36 |
| G2/M | 14.01 ± 2.39 | 6.46 ± 3.93 | 7.56 ± 4.23 | 2.04 ± 3.79 |

An asterisk (*) indicates significance with *p*-value < 0.05 when compared to cells propagated in complete growth medium.

Table S3: illustration of apoptosis, necrosis and viable cells after glutamine deprivation.

| **Condition** | **MCF-7** | **MDA-MB-231** | **MCF10-A** | **BT-20** |
| --- | --- | --- | --- | --- |
| **24 h Medium only** | **Average ± SD (%)** | **Average ± SD (%)** | **Average ± SD (%)** | **Average ± SD (%)** |
| Early apoptosis | 1.23 ± 1.4 | 1.96 ± 0.04 | 0.75 ± 0.63 | 0.99 ± 0.34 |
| Late apoptosis | 1.05 ± 2.63 | 2.18 ± 0.04 | 3.89 ± 0.96 | 0.39 ± 0.44 |
| Viable cells | 96.31 ± 2.7 | 95.42 ± 1.16 | 93.47 ± 1.03 | 96.29 ± 1.25 |
| Necrosis | 2.41 ± 1.09 | 0.73 ± 1.16 | 1.89 ± 0.97 | 2.33 ± 0.53 |
| **24 h without Glutamine** | **Average ± SD (%)** | **Average ± SD (%)** | **Average ± SD (%)** | **Average ± SD (%)** |
| Early apoptosis | 3.49 ± 1.78 | 3.72 ± 0.54 | 1.68 ± 0.3 | 1.81 ± 1.36 |
| Late apoptosis | 3.00 ± 1.7 | 3.49 ± 1.92 | 3.82 ± 1.5 | 0.53 ± 0.30 |
| Viable cells | 87.61 ± 4.87* | 88.14 ± 1.92* | 92.1 ± 1.1 | 86.86 ± 4.97* |
| Necrosis | 5.69 ± 1.9 | 4.29 ± 2.27 | 2.9 ± 1.84 | 10.79 ± 2.75 |
| **48 h Medium only** | **Average ± SD (%)** | **Average ± SD (%)** | **Average ± SD (%)** | **Average ± SD (%)** |
| Early apoptosis | 0.21 ± 2.31 | 1.01 ± 1.49 | 0.93 ± 2.05 | 1.39 ± 0.43 |
| Late apoptosis | 1.74 ± 0.88 | 1.03 ± 1.99 | 0.41 ± 0.49 | 0.96 ± 0.34 |
| Viable cells | 95.75 ± 3.96 | 97.58 ± 2.17 | 97.29 ± 4.14 | 96.05 ± 0.98 |
| Necrosis | 2.3 ± 2.97 | 1.06 ± 0.89 | 1.37 ± 2.51 | 4.6 ± 0.60 |
| **48 h without Glutamine** | **Average ± SD (%)** | **Average ± SD (%)** | **Average ± SD (%)** | **Average ± SD (%)** |
| Early apoptosis | 9.87 ± 0.45* | 3.06 ± 0.17 | 2.33 ± 0.9* | 0.71 ± 0.21 |
| Late apoptosis | 9.25 ± 0.40* | 2.71 ± 0.37 | 5.38 ± 2.24* | 0.45 ± 0.19 |
| Viable cells | 78.91 ± 1.53* | 89.33 ± 1.27* | 88.24 ± 1.26* | 85.6 ± 2.93* |
| Necrosis | 2.97 ± 1.19 | 4.9 ± 1.17* | 9.04 ± 2.56* | 13.23 ± 4.28* |
| **72 h Medium only** | **Average ± SD (%)** | **Average ± SD (%)** | **Average ± SD (%)** | **Average ± SD (%)** |
| Early apoptosis | 0.1 ± 1.19 | 0.3 ± 0.51 | 0.88 ± 0.41 | 1.69 ± 0.86 |
| Late apoptosis | 0.81 ± 1.96 | 0.69 ± 2.06 | 0.89 ± 1.84 | 1.76 ± 0.77 |
| Viable cells | 97.3 ± 3.09 | 98.65 ± 2.14 | 96.6 ± 2.37 | 96.57 ± 1.58 |
| Necrosis | 0.87 ± 1.52 | 0.99 ± 1.63 | 1.63 ± 0.56 | 3.98 ± 0.15 |
| **72 h without Glutamine** | **Average ± SD (%)** | **Average ± SD (%)** | **Average ± SD (%)** | **Average ± SD (%)** |
| Early apoptosis | 3.39 ± 0.5* | 0.56 ± 0.43 | 0.75 ± 0.35 | 0.97 ± 0.31 |
| Late apoptosis | 3.06 ± 0.85* | 0.49 ± 0.53 | 2.51 ± 1.05 | 0.58 ± 0.34 |
| Viable cells | 91.84 ± 1.74 | 89.21 ± 1.22 | 93.86 ± 4.23 | 90.95 ± 0.56 |
| Necrosis | 5.7 ± 0.52* | 9.73 ± 0.39* | 2.5 ± 2.19 | 7.74 ± 1.06 |
| **96 h Medium only** | **Average ± SD (%)** | **Average ± SD (%)** | **Average ± SD (%)** | **Average ± SD (%)** |
| Early apoptosis | 1.55 ± 0.5 | 1.36 ± 1.42 | 1.36 ± 0.25 | 0.83 ± 0.45 |
| Late apoptosis | 1.36 ± 0.9 | 1.71 ± 2.23 | 2.91 ± 2.45 | 0.96 ± 0.52 |
| Viable cells | 96.3 ± 2.27 | 97.03 ± 3.48 | 93.77 ± 4.97 | 96.22 ± 1.1 |
| Necrosis | 0.79 ± 1.2 | 0.9 ± 1.13 | 1.96 ± 4.17 | 6.99 ± 0.44 |
| **96 h without Glutamine** | **Average ± SD (%)** | **Average ± SD (%)** | **Average ± SD (%)** | **Average ± SD (%)** |
| Early apoptosis | 1.68 ± 2.23 | 6.25 ± 3.27 | 1.26 ± 1.24 | 0.92 ± 1.23 |
| Late apoptosis | 1.75 ± 2.77 | 6.22 ± 1.92 | 3.06 ± 1.94 | 0.65 ± 0.28 |
| Viable cells | 93.81 ± 4.39 | 75.49 ± 1.64* | 93.67 ± 4.67 | 90.61 ± 2.3 |
| Necrosis | 2.45 ± 1.05* | 12.08 ± 1.56 | 2.1 ± 1.76 | 8.07 ± 1.89 |
| **ESE-ol treated 48h** | **Average ± SD (%)** | **Average ± SD (%)** | **Average ± SD (%)** | **Average ± SD (%)** |
| Early apoptosis | 56.3 ± 0.16* | 41.2 ± 0 | 39.47 ± 0.35 | 28.36 ± 0.37 |
| Late apoptosis | 31.2 ± 4.69* | 42.32 ± 2.28 | 10.78 ± 0.62 | 63.89 ± 0.02 |
| Viable cells | 3.14 ± 2.82* | 3.69 ± 4.00 | 3.45 ± 2.7 | 1.39 ± 1.37 |
| Necrosis | 9.36 ± 2.58 | 12.79 ± 2.7 | 46.3 ± 2.52 | 6.36 ± 1.41 |

An asterisk (*) indicates significance with *p*-value < 0.05 when compared to cells propagated in complete growth medium.
